# Predicting the path to guided ICBT success across referral pathway for moderate depression and anxiety disorders

**DOI:** 10.1016/j.invent.2026.100907

**Published:** 2026-01-19

**Authors:** Jill Bjarke, Rolf Gjestad, Tine Nordgreen

**Affiliations:** a Research Centre for Digital Mental Health Services, Division of Psychiatry, Haukeland University Hospital, Bergen, Norway; b Department of Global Public Health and Primary Care, University of Bergen, Bergen, Norway; c Research Department, Division of Psychiatry, Haukeland University Hospital, Bergen, Norway; d Centre for Research and Education in Forensic Psychiatry, Haukeland University Hospital, Bergen, Norway; e Centre for Crisis Psychology, Faculty of Psychology, University of Bergen, Bergen, Norway

**Keywords:** Guided internet-delivered cognitive behavioral therapy, Depression, Anxiety disorders, General practitioner referral, Self-referral, Routine clinical practice, Prediction analyses

## Abstract

**Background:**

Depression and anxiety disorders are highly prevalent and burdensome conditions. Guided internet-delivered cognitive behavioral therapy (guided ICBT) is an effective treatment option, yet outcome prediction has shown inconsistent results. By analyzing patient-, treatment-, and system-related predictors within a single integrative framework, we aim to support a more nuanced understanding of what works for whom in which context.

**Method:**

Naturalistic, open predictor-study in routine care comparing symptom reduction from pre-treatment (Pre) to post-treatment (Post), and from Pre to 6-month follow-up (FU), across general practitioner (GP) and self-referral pathways for depression and anxiety disorders. Patients self-reported symptoms Pre, Post, and FU. Symptom severity and change over time were assessed transdiagnostically applying a harmonized outcome score. Patient-related predictors were gender, age, education, employment status, duration of illness, and social support, while treatment-related predictors were treatment credibility and self-efficacy. The system-related predictor was referral pathways.

**Results:**

In the total sample (*N* = 460, GP = 305, Self-referred = 155), almost all patient-, treatment- and system-related predictors were associated with symptom reduction over time measured at Post and FU. Referral pathway statistically significant moderated the associations between most predictors and symptom reduction.

**Conclusion:**

Using a novel integrative approach, we identified unique adjusted associations between predictors across patient-, treatment-, and system-related predictors and symptom reduction in guided ICBT. Referral pathway moderated their relevance, underscoring the importance of contextual factors in shaping treatment response in routine care.

## Highlights

• Referral pathway predicted and moderated symptom reduction in guided ICBT.
• Self-referred patients improved more than GP-referred at Post and 6-month follow-up.
• Predictive value of pre-treatment factors varied by referral pathway.
• Treatment credibility and self-efficacy predicted short- and long-term outcomes.
• Lower social support was associated with greater symptom reduction in both groups.

## 1 Introduction

Depression and anxiety disorders are highly prevalent and costly disorders, both on an individual and a societal level (GBD 2019 Mental Disorders Collaborators, 2022). Guided internet-delivered cognitive behavioral therapy (guided ICBT) is an effective intervention for treating depression and anxiety in adults. Guided ICBT has equal effect as face-to-face CBT (Hedman-Lagerlöf et al., 2023), and for many conditions, there is now even more evidence supporting internet-delivered treatments than face-to-face formats in terms of study size and data quality (Andersson, 2024). Guided ICBT also works well in routine care settings (Etzelmueller et al., 2020). Despite the documented efficacy of guided ICBT, not all patients respond equally well to the treatment (Rozental et al., 2019; Andersson et al., 2019). This has led to an increased focus on understanding what works for whom in which context. Recent systematic reviews of internet-delivered interventions have identified a wide range of predictors of treatment response for patients with depression and anxiety disorders (Haller et al., 2023; Sextl-Plötz et al., 2024). Yet, to our knowledge, no previous studies have examined patient-, treatment-, and system-related predictors within a single framework, nor explored the interplay between these domains in shaping treatment response.

### 1.1 Patient-related predictors

Pre-treatment symptom severity has in systematic reviews, been associated with both greater symptom reduction and higher symptom levels post-treatment (Haller et al., 2023; Sextl-Plötz et al., 2024). This association has also been confirmed using machine learning techniques (ML), which identified higher pre-treatment severity as a key predictor of positive treatment response (Hornstein et al., 2021), thereby strengthening the robustness of this finding across analytic approaches.

The demographic predictors gender, age, education and duration of illness have in systematic reviews shown generally weak associations with treatment outcomes (Haller et al., 2023; Sextl-Plötz et al., 2024). Patient-level meta-analyses found a small added effect of being a woman in depression treatment (Andersson et al., 2019), while another meta-analyses identified being male as a predictor of non-response across diagnoses (Rozental et al., 2019). Evidence from routine care studies have also been mixed. For example, in guided ICBT for health anxiety, higher age was associated with greater likelihood of treatment completion (Österman et al., 2024), while higher education has been linked to lower initial symptom severity but less improvement (Edmonds et al., 2018). Notably, a ML-study on guided-internet intervention, found that not having a university degree predicted greater symptom improvement (Hornstein et al., 2021). Duration of illness has been included as a predictor of symptom reduction in routine care studies, but it is rarely retained in final models and has not consistently emerged as a significant factor in treatment outcomes (El Alaoui et al., 2015a, El Alaoui et al., 2016; Hedman et al., 2015).

Employment status is also a frequently examined but inconclusive predictor, where systematic reviews report inconsistent and non-significant associations with treatment outcomes (Haller et al., 2023; Sextl-Plötz et al., 2024). Still, several individual routine care studies report that being employed, particularly working full-time, is associated with lower post-treatment symptom severity (El Alaoui et al., 2016; Hedman et al., 2012; Niles et al., 2021).

Social support has rarely been examined as a predictor in internet-delivered interventions. A systematic review identified only three studies addressing this variable, most with high risk of bias and inconclusive findings (Haller et al., 2023). A large routine care study found that higher availability of supportive social relationships at pre-treatment predicted significantly greater symptom reduction following a 12-week guided ICBT intervention for depression (Hallgren et al., 2017).

### 1.2 Treatment-related predictors

Treatment-related predictors refer to psychological factors that reflect the patient's perception of, and confidence in the therapeutic process. Treatment credibility, defined as how convincing, appropriate, and reasonable a patient perceives the treatment to be (Kazdin, 1979), has emerged as a consistent, albeit modest, predictor and moderator for symptom reduction in synthesized research (Haller et al., 2023; Kumpasoğlu et al., 2025; Pontén et al., 2024). In routine care studies, higher treatment credibility has been found to predict greater symptom reduction and lower post-treatment symptom levels of depression (El Alaoui et al., 2016). In contrast, its predictive value for long-term change in anxiety disorder appears limited when controlling for pre-treatment illness severity (El Alaoui et al., 2015b).

Self-efficacy, defined as the individual's beliefs in their capacity to carry out specific behaviors (Zulkosky, 2009), is conceptualized in the present study as confidence in managing the specific tasks and challenges involved in guided ICBT. Given that guided ICBT demands personal responsibility and sustained motivation due to reduced therapeutic contact (Schønning and Nordgreen, 2021), self-efficacy may be particularly relevant for successful treatment engagement and outcome. However, self-efficacy has shown inconsistent results as a predictor in systematic reviews (Haller et al., 2023; Behr et al., 2025), yet, it is suggested to play a mediating role in treatment outcomes in internet-delivered interventions (Behr et al., 2025). In a routine care study on guided ICBT for anxiety disorders, higher levels of self-efficacy both predicted and moderated symptom improvement (Schønning and Nordgreen, 2021).

### 1.3 System-related predictors

Referral pathway through which patients access treatment has emerged as a potentially relevant predictor, warranting further investigation. Studies directly comparing the effectiveness of internet-delivered interventions between GP-referrals versus self-referral are limited, with mixed results. Some report better outcomes for GP-referrals (Mataix-Cols et al., 2006), others for self-referrals (Bjarke et al., 2025; Staples et al., 2022) and some find no significant difference in effect between the two referral pathways (Arndt et al., 2020). In a subgroup analysis of a meta-analysis of guided ICBT in routine care, studies recruiting participants from the general population based on open recruitment strategies like web-based advertisements (interpreted as self-referral) showed significantly larger treatment effects for individuals with depression than studies recruiting via GPs, other healthcare providers or mixed pathways. However, heterogeneity was high, and no such pattern was found among individuals with anxiety disorder (Etzelmueller et al., 2020).

Prediction studies have begun to explore whether referral pathway itself may influence treatment outcomes (Hornstein et al., 2021; White, 2009). In a study on children and adolescents with major depressive disorder (Emslie et al., 2008; Kennard et al., 2008), referral pathway was not a significant predictor (White, 2009). In contrast, in a ML-study, referral pathway was an influential predictor in a guided internet-delivered intervention for depression and anxiety, with self-referral predicting higher likelihood of improvement (Hornstein et al., 2021).

These mixed findings suggest that referral pathway may reflect deeper contextual differences in how patients access care, including variations in motivation and perceived autonomy in treatment choice (Titov et al., 2018). Self-referral is an established pathway to ICBT-clinics in several countries (Titov et al., 2018; Folker et al., 2018), yet to our knowledge, no previous study has examined referral pathway as a potential moderating factor on treatment outcomes in routine clinical care.

### 1.4 Integrative rationale

While previous studies have examined predictors within isolated domains, their findings often point in divergent directions, depending on context, sample, and methodology. By analyzing a large, naturalistic sample from routine care, and modeling patient-, treatment-, and system-related predictors concurrently within a single adjusted framework, this study aims to contribute to a more nuanced understanding of who benefits from guided ICBT, and under which circumstances. This aligns with calls for research that goes beyond effectiveness to examine system- and context-related factors in routine care settings (Etzelmueller et al., 2020).

#### 1.4.1 Aim

The primary aim was to investigate the unique and adjusted contributions of patient-related predictors (age, gender, education level, employment status, duration of illness, social support), treatment-related predictors (treatment credibility, self-efficacy), and system-related predictors (referral pathway) to symptom reduction over time in guided ICBT for depression and anxiety disorders. A secondary aim was to explore whether the predictive value of these factors varied by referral pathway.

## 2 Method

### 2.1 Design

This open naturalistic longitudinal study was conducted at the eCoping clinic, a specialized routine mental health care clinic at Haukeland University Hospital in Bergen, Norway. We compared two groups of patients: those referred by general practitioners (GP-referred) and those who self-referred to guided internet-delivered cognitive behavioral therapy (guided ICBT) for moderate depression, panic disorder (PD), or social anxiety disorder (SAD). The study presents data collected between September 2014 and May 2019.

### 2.2 Ethics

The study, *eMeistring- på nett med fastlegen* (the eCoping-study), was approved by the Regional Committee for Medical Research Ethics (REK) 2014/2175. The authors assert that all procedures contributing to this work comply with the ethical standards of the relevant national and institutional committees on human experimentation, and with principles of the Declaration of Helsinki (World Medical Association, 2018).

### 2.3 Setting

Since 2013, the eCoping clinic has provided guided ICBT as part of its routine care for panic disorder (PD) (Nordgreen et al., 2018a) and social anxiety disorder (SAD) (Nordgreen et al., 2018b), while guided ICBT for moderate depression was introduced in 2015 (Nordgreen et al., 2019). Treatment lasted up to 14 weeks for all diagnoses, with eight modules for depression and nine for both anxiety disorders. Patients progressed through the modules sequentially, spending about 7–10 days per module, each taking roughly 45 min to complete. Therapists provided individualized feedback and guidance asynchronously after each module or at least weekly, and introduced the next module as needed. Therapist time averaged 10–30 min per patient per week. Further study design and treatment results have previously been described in detail (Nordgreen et al., 2018a, Nordgreen et al., 2018b, Nordgreen et al., 2019). For the present study, we use data collected continuously throughout the modules, but present the results from pre-treatment, post-treatment and 6-month follow-up assessments. We also present pre-treatment demographic characteristics, level of social support, self-efficacy and treatment credibility across referral pathways.

#### 2.3.1 Sample and procedure

A specialist in clinical psychology reviewed all referrals regardless of referral pathway in accordance with national priority guidelines for determining eligibility for specialized care treatment (Helsedirektoratet, 2015). Inclusion criteria for all patients were: 1) being 18 years of age or older, 2) diagnosed either with major depressive episode, social anxiety disorder, or panic disorder according 3) if self-reported antidepressants use, being on a stable dosage over the previous four weeks, 4) fluent in oral and written Norwegian. Exclusion criteria for all patients were: 1) current suicidal ideation, 2) current psychosis, 3) current substance abuse, 4) using benzodiazepines daily, 5) immediate need of other treatment, 5) no access to the internet. All study-participants signed a written informed consent. Patients meeting the criteria for treatment received a scheduled appointment for a face-to-face consultation.

All patients granted therapy participated in the face-to-face consultation that included a diagnostic interview using the Mini-International Neuropsychiatric Interview (MINI) (Sheehan et al., 1998). Treatment program allocation was based on the results of the MINI assessment alongside the therapists' clinical judgment. Participants were diagnosed with either moderate depression, PD, or SAD.

### 2.4 Measures

#### 2.4.1 Patient-related predictors

All self-reported demographic questionnaires were administered via the internet and collected pre-treatment. We measured basic demographic characteristics using a questionnaire specifically designed for this study. It included questions about gender, assessed via a binary item with the response options *woman* and *man*. Additional questions covered age, educational level, employment status, and perceived level of social support. Participants were also asked how long they had experienced symptoms related to their current disorder.

We measured social support with a questionnaire covering different aspects of functional and structural support. There is no universally accepted gold-standard instrument for measuring social support, and existing tools vary in length and conceptual focus. To ensure relevance and feasibility for the study context, the questionnaire was designed by the research group affiliated with the eCoping study. The questionnaire entails 7 questions where higher scores indicate higher level of social support (total score range: 0–27) (Appendix 1). Given support for a unidimensional factor structure, reporting the total score is most appropriate and reflects the construct of social support. Internal consistency measured by Cronbach's alpha, yielded 0.81 for GP-referrals and 0.79 for self-referred patients.

#### 2.4.2 Treatment-related predictors

We measured treatment self-efficacy with a modified version of the Bergen Genetic Counseling Self-Efficacy Scale (Bjorvatn et al., 2008). The original scale was developed by a panel of medical experts using Bandura's Guide for Constructing Self-efficacy Scales (Bandura, 2006), and piloted in a clinical setting (Bjorvatn et al., 2008). The modified version was adapted to fit a guided internet-delivered therapy format. The scale comprises 15 items describing tasks and challenges that are likely to occur during guided ICBT, and the individual's beliefs that they will be able to cope with these. Each item was rated on a scale from 0 to 10 (0 = cannot do at all, 10 = can do without difficulty), with a total range of 0–150, where higher scores indicate higher level of self-efficacy. Internal consistency measured with the Cronbach's alpha yielded 0.87 for GP-referred participants and 0.81 for self-referred.

We measured treatment credibility after completion of the first treatment module to ensure that all participants had a shared experience of the treatment format and content, reducing variability due to differing intervals between initial interview and treatment start. We used a modified version of the Credibility and Expectancy Scale (CEQ) (Borkovec and Nau, 1972). The modified version was adapted to fit a guided internet-delivered therapy format. The scale comprises 5 items, each item was rated on a scale from 0 to 10 (0 = cannot do at all, 10 = can do without difficulty), total range was 0–50, where higher scores indicate higher treatment credibility. Internal consistency measured with the Cronbach's alpha yielded 0.89 for GP-referred participants and 0.93 for self-referred.

#### 2.4.3 System-related predictors

Patients referred by GPs were evaluated by their doctors for symptom severity to determine the need for specialized care services; if deemed necessary, the GP authored a referral to the eCoping clinic. Patients who self-referred sent an email with their contact details to an address available on the eCoping website. Subsequently, an eCoping therapist conducted a clinical interview by telephone to assess symptom severity and the treatment's relevance. A summary of the interview was generated as a self-referral.

#### 2.4.4 Symptom specific outcome measures

Treatment outcomes were assessed in terms of symptom reduction, defined as the change in symptom severity from Pre-treatment to Post-treatment, and from Pre-treatment to 6-month Follow-up, and collected at the corresponding assessment points.

We measured depression with Montgomery Åsberg Depression Rating Scale, Self-rating version (MADRS-S) (Svanborg and Åsberg, 1994). This scale includes 9 items, each scored on a Likert scale ranging from 0 to 6, resulting in a total score between 0 and 54, where higher scores reflect greater symptom severity. The MADRS-S has demonstrated sensitivity to change (Fantino and Moore, 2009; Svanborg and Åsberg, 1994), and strong correlations between expert ratings and self-reports (Svanborg and Åsberg, 1994). Internal consistency measured with the Cronbach's alpha yielded 0.77 for participants with GP-referral and 0.82 for self-referred participants.

We measured panic disorder (PD) with the Body Sensation Questionnaire (BSQ) (Chambless, 1984). The BSQ consists of 16 items, each rated on a 5-point Likert scale, yielding a total score between 16 and 80. Higher scores indicate greater fear and sensitivity to bodily sensations typically associated with autonomic nervous system arousal. The scale has been found sensitive to symptom changes during treatment (Chambless, 1984). Internal consistency measured with the Cronbach's alpha yielded 0.84 for participants with GP-referral and 0.88 for self-referred participants and showed good internal consistency reliability.

We measured social anxiety disorder (SAD) with the Social Phobia Scale (SPS) (Mattick and Clarke, 1998). The SPS measures social phobia and the distress of being observed or watched while performing daily activities in the presence of others (Mattick and Clarke, 1998). It comprises 20 items rated on a 5-point Likert scale, producing a total score ranging from 0 to 100, with higher scores reflecting greater anxiety about being observed or scrutinized. The scale has demonstrated good reliability and validity (Brown et al., 1997; Mattick and Clarke, 1998), along with discriminant validity in differentiating individuals with SAD from healthy controls and those with other anxiety disorders (Mattick and Clarke, 1998). Internal consistency measured with the Cronbach's alpha yielded 0.91 for GP-referrals and 0.93 for self-referred participants.

To increase power and to allow comparison across diagnoses, we combined the outcome measures for the three treatment modalities into one harmonized outcome score (see formula). This ensures comparability across different measurement scales and normalizes the total score by first subtracting the minimum possible value, then dividing by the range (maximum possible value minus minimum), and finally multiplying by 100. The harmonized score combines specific symptom measures into a single index representing overall symptom burden. Such harmonization increases the overall sample size and enhances the statistical power of the analyses (Zantvoort et al., 2024). To assess the appropriateness of the harmonization, we calculated percentage reductions in the diagnosis-specific symptom scores for each disorder (Appendix 2). The comparable patterns of symptom reductions support the methodological validity of combining these into a single transdiagnostic outcome score. Internal consistency using Cronbach's alpha could not be calculated for the harmonized outcome measure because the study design allowed for missing data at item level. Participants only completed outcome measures relevant to their specific diagnoses.

$$H=\frac{\mathrm{scor}-\mathrm{MIN}}{\mathrm{MAX}-\mathrm{MIN}}*100$$

### 2.5 Statistics

Statistical analyses were conducted using SPSS version 29 (IBM Corp, 2023), including descriptive statistics (mean, SD, percent) and bivariate analyses (cross tabulation, *t*-test). Mplus version 8.11 (Muthén and Muthén, 2024) used for multilevel (linear mixed model) analyses of the outcome over time at the within level, regressed on the between level predictors (Hox et al., 2018). A random intercept fixed slope model was specified with Modules, Pre-treatment, Post-treatment, and 6-month Follow-up measurements with dummy variables. The intercept factor was centered to Pre-treatment assessment, representing changes from pre-treatment to all consecutive Modules, Post-treatment, and Follow-up. Only results from Pre-treatment to Post-treatment (Pre-Post) and from Pre-treatment to the Follow-up are presented (Pre-FU) in the results. The continuous predictor variables (age, duration of illness, credibility, social support, self-efficacy) were mean centered to ease the interpretation of the LMM results, and to reduce potential multicollinearity in the interaction analyses (Hox et al., 2018).

First, the predictors were added to the model to test the predictive associations with changes over time, but also with pre-treatment level. After all, this is the starting point of change (primary aim). Second, to test whether these relations between predictors and outcome differed for the referral groups (contextual factor), a multi-sample two-level analysis was used. Differences in predictions were tested with model constraints (secondary aim).

The estimator was robust maximum likelihood (MLR) for adjustment for potential non-normality (Kline, 2016). Confidence levels (CI) were reported as 95% CI. Small amount of missing data were observed in the predictors and SPSS was used for Expectation Maximization (EM) data imputation under the missing at random (MAR) assumption (Enders, 2010). The exception was information about employment status, which should not be imputed. Therefore, to use all cases for the analyses, and avoid listwise deletion of data, the variable employment status was included in the model by declaring its variance. To compare the relative magnitude of the predictors we analyzed these models based on z-score transformed predictor- and outcome-variables.

## 3 Results

### 3.1 Pre-treatment patient- and treatment related predictors

In the total sample (*N* = 460), about six out of ten patients were women (see Table 1). The mean age was just above thirty years with considerable individual variability. About 14% of the sample was educated at the tertiary level, and about half the sample were employed. The mean duration of illness was ten years. Table 1 also includes information about treatment credibility, social support and self-efficacy.

**Table 1:** Descriptive information about patient- and treatment related predictors (*N* = 460).

	GP-referred		Self-referred		Total		
	*N* = 305		*N* = 155		N = 460		*P*
Gender: n/N (%)							0.054
Woman	176	(60.9)	106	(70.2)	282/440	(64.1)	
Man	113	(39.1)	45	(29.8)	158/440	(35.9)	
Age at pretreatment, mean (SD)	32.7	(11.4)	31.9	(10.3)	32.5	(11.0)	0.122
Education: n/N (%)							<0.001
Primary level	46	(16.0)	14	(9.3)	60/439	(13.7)	
Secondary level	143	(49.7)	50	(33.1)	193/439	(44.0)	
Tertiary level	99	(34.4)	87	(57.6)	63.3	(11.7)	
Employment							
Years with complaints: Mean (SD)	10.35	(9.62)	9.80	(9.43)	10.02	(9.5)	0.538
Treatment program: N (%)							0.002
Depression	53	(17.4)	48	(31.0)	101	(21.7)	
Panic disorder	116	(38.0)	56	(36.1)	172	(37.4)	
Social anxiety disorder	136	(44.6)	51	(32.9)	187	(40.7)	
Social support	21.2	(4.4)	22.3	(4.3)	21.6	(4.4)	0.016
Treatment credibility	7.1	(1.5)	7.5	(1.2)	7.2	(1.4)	<0.001
Self-efficacy	7.6	(1.2)	7.7	(1.4)	7.6	(1.3)	0.179

N: sample size for that measure, n: number of participants with that characteristic.

SD: standard deviation.

### 3.2 Relations between patient-, treatment, and system-related predictors and outcome

#### 3.2.1 Patient related predictors

Adjusted associations at pre-treatment (Pre), showed that higher symptom levels were linked to lower age, being a woman, lower levels of social support, and longer duration of illness for the total sample. Statistically significant associations for the GP-referred indicated that higher pre-treatment symptoms were related to being younger and reporting lower levels of social support. Associations for the self-referred revealed that higher Pre-treatment symptoms were connected to lower educational levels and lower levels of social support.

The results related to the primary aim investigating predictors of symptom changes over time, showed that from Pre to post treatment (Post), stronger symptom reduction was associated with younger age, being a woman, shorter duration of illness, being employed and lower levels of social support (Table 2). The results from Pre to 6-month Follow-up (FU) were similar, with one exception: stronger symptom reduction from Pre to FU was linked to lower levels of education (Table 2).

**Table 2:** LMM adjusted results between patient-, treatment, and system-related predictors and pre-symptom levels and symptom reduction from Pre to Post, and from Pre to FU.

				95% CI	
Predictors	*b*	*β*	*p*	Low	High
GP (Intercept)	47.56		<0.001	43.65	51.46
Self-referred	−1.54	−0.05	0.354	−4.80	1.72
Age ^c^	−0.38	−0.21	0.000	−0.53	−0.23
Woman	3.35	0.08	0.037	0.21	6.50
Education	−2.25	−0.08	0.068	−4.66	0.17
Employed	−1.80	−0.04	0.281	−5.06	1.47
Social support^c^	−0.87	−0.19	0.000	−1.24	−0.51
Duration of illness^c^	0.26	0.12	0.004	0.08	0.43
Credibility^a^, ^c^	1.28	0.09	0.033	0.11	2.46
Self-efficacy^c^	−0.42	−0.03	0.520	−1.72	0.87
Post – Pre	−9.57		<0.001	−11.01	−8.14
Post*Self-referred	−2.79	−0.07	<0.001	−3.08	−2.50
Post*Age	0.09	0.05	0.010	0.02	0.15
Post*Woman	−3.77	−0.09	<0.001	−4.20	−3.34
Post*Education	−0.16	−0.01	0.349	−0.48	0.17
Post*Employed	−6.37	−0.16	<0.001	−6.69	−6.06
Post*Social support	0.13	0.03	<0.001	0.08	0.18
Post*Duration of illness	0.06	0.03	0.032	0.01	0.12
Post*Credibility	−3.23	−0.23	<0.001	−3.31	−3.15
Post*Self-efficacy	−0.60	−0.04	<0.001	−0.77	−0.44
FU - Pre	−15.55		<0.001	−17.27	−13.84
FU*Self-referred	−2.80	−0.07	<0.001	−3.11	−2.48
FU*Age	0.16	0.09	<0.001	0.13	0.20
FU*Woman	−1.42	−0.03	<0.001	−1.78	−1.07
FU*Education	0.69	0.02	<0.001	0.33	1.06
FU*Employed	−7.26	−0.18	<0.001	−7.54	−6.97
FU*Social support	0.36	0.08	<0.001	0.29	0.42
FU*Duration of illness	0.17	0.08	<0.001	0.13	0.20
FU*Credibility	−4.57	−0.33	<0.001	−4.64	−4.49
FU*Self-efficacy	0.95	0.06	<0.001	0.74	1.16

β: Standardized regression by z-transforming predictor- and outcome variables Pre: pre-treatment, Post: post-treatment, FU: 6-month follow-up.

Intercept: GP-referred men, with primary education level, being unemployed, and at mean levels of age, duration of illness, Credibility, Social support, and Self-efficacy.

a Treatment credibility.

c Mean centered variable.

The secondary aim, which explored differences between the referral pathways, was addressed through a multi-sample model. The results showed some statistically significant differences when differentiating the relations for patient- and treatment-related predictors at the system-level between self-referred and GP referred patients from Pre to Post and from Pre to FU (Table 3). First, almost all statistically significant relations found in the total sample were also statistically significant for GP-referred patients, however, with some differences in the magnitude. Among GP-referred patients, stronger symptom reduction from Pre to Post was associated with being a woman, younger age, higher educational levels, shorter duration of illness, being employed, and lower levels of social support. From Pre to FU, stronger symptom reduction in this group was associated with being a man, being younger, higher levels of education, shorter duration of illness, being employed, and lower levels of social support. Among the self-referred patients, we found fewer statistically significant associations. From Pre to Post, stronger symptom reduction was associated with being younger, being a woman, and having lower levels of social support.

**Table 3:** LMM adjusted results of prediction of level and change in symptoms from Pre to Post, and from Pre to FU in the two referral-groups: General practitioner referral (GP) and Self-referral (SELF).

	GP					SELF					Difference	
Parameter	*b*	*β*	*p*	95% CI		*b*	*β*		95% CI			
				Lower	Upper			*p*	Lower	Upper	∆	*p*
Intercept	45.94		<0.001	41.57	50.32	49.91		<0.001	42.53	57.28	3.96	0.365
Age^c^	−0.39	−0.22	<0.001	−0.57	−0.22	−0.27	−0.15	0.059	−0.54	0.01	0.13	0.438
Woman	2.95	0.07	0.111	−0.68	6.58	3.67	0.09	0.181	−1.70	9.03	0.72	0.828
Education	−0.68	−0.02	0.629	−3.42	2.07	−4.53	−0.15	0.028	−8.58	−0.48	−3.86	0.122
Employment	−1.85	−0.05	0.335	−5.63	1.92	−1.86	−0.05	0.530	−7.66	3.94	−0.00	0.999
Social support^c^	−0.86	−0.19	<0.001	−1.29	−0.44	−0.73	−0.17	0.018	−1.34	−0.13	0.13	0.729
Duration (of illness)^c^	0.16	0.08	0.118	−0.04	0.37	0.22	0.11	0.157	−0.09	0.53	0.06	0.756
Credibility^c^	1.25	0.10	0.071	−0.11	2.61	−0.09	−0.01	0.936	−2.27	2.09	−1.34	0.305
Self-efficacy^c^	−0.36	−0.02	0.687	−2.11	1.39	−0.61	−0.05	0.500	−2.38	1.16	−0.25	0.845
Post	−7.97		<0.001	−10.23	−5.71	−13.87		<0.001	−16.23	−11.52	−5.91	<0.001
Post*Age	0.09	0.05	<0.001	0.04	0.14	0.11	0.06	0.021	0.02	0.20	0.02	0.718
Post*Woman	−1.78	−0.04	0.001	−2.80	−0.75	−8.36	−0.21	<0.001	−9.03	−7.70	−6.59	<0.001
Post*Education	−1.38	−0.05	<0.001	−2.07	−0.70	−0.23	−0.01	0.790	−1.92	1.46	1.15	0.216
Post*Employment	−8.98	−0.22	<0.001	−9.18	−8.77	−0.87	−0.02	0.554	−3.74	2.00	8.11	<0.001
Post*Social support	0.13	0.03	0.001	0.05	0.22	0.35	0.08	<0.001	0.17	0.53	0.21	0.035
Post*Duration	0.21	0.10	<0.001	0.17	0.24	−0.12	−0.06	0.067	−0.25	0.01	−0.33	<0.001
Post*Credibility^a^	−3.43	−0.26	<0.001	−3.53	−3.32	−2.04	−0.13	<0.001	−2.52	−1.56	1.38	<0.001
Post*Self-efficacy	−1.06	−0.06	<0.001	−1.21	−0.90	−0.27	−0.02	0.430	−0.94	0.40	0.78	0.026
FU	−12.90		<0.001	−15.00	−10.81	−22.06		<0.001	−24.93	−19.17	−9.15	<0.001
FU*Age	0.09	0.05	<0.001	0.05	0.13	0.28	0.15	<0.001	0.17	0.38	0.19	0.001
FU*Woman	1.17	0.03	0.005	0.35	1.98	−4.90	−0.12	<0.001	−5.57	−4.23	−6.06	<0.001
FU*Education	−0.78	−0.03	0.005	−1.33	−0.23	0.61	0.02	0.433	−0.91	2.12	1.39	0.091
FU*Employment	−11.23	−0.28	<0.001	−11.44	−11.01	0.66	0.02	0.356	−0.75	2.07	11.89	<0.001
FU*Social support	0.31	0.07	<0.001	0.23	0.38	0.69	0.16	<0.001	0.57	0.80	0.38	<0.001
FU*Duration	0.25	0.12	<0.001	0.22	0.28	0.03	0.02	0.688	−0.13	0.20	−0.22	0.013
FU*Credibility	−5.43	−0.41	<0.001	−5.50	−5.35	−4.10	−0.27	<0.001	−4.44	−3.76	1.33	<0.001
FU*Self-efficacy	1.47	0.09	<0.001	1.27	1.67	0.52	0.04	0.187	−0.25	1.30	−0.94	0.021

β: Standardized regression by z-transforming predictor- and outcome variables.

Post: post-treatment, FU: 6-month follow-up.

Intercept: Reference: GP-referred men, at mean level of age, duration of illness, Credibility, Social support, Self-efficacy, with a primary education level, and being unemployed.

a Treatment credibility.

c Mean centered variable.

#### 3.2.2 Treatment related predictors

Association at pre-treatment showed that higher symptom levels were related to higher levels of treatment credibility for the total sample.

Primary aim: stronger symptom reduction from Pre to Post was associated with higher levels of treatment credibility and self-efficacy (Table 2). When examining symptom reduction from Pre to FU, stronger reduction was linked to lower levels of self-efficacy and to higher levels of treatment credibility.

Secondary aim: Stronger symptom reduction from Pre to Post was related to higher treatment credibility and self-efficacy among GP-referred patients, while stronger symptom reduction from Pre to FU was associated with higher treatment credibility but lower levels of self-efficacy for these same patients (Table 3). Stronger symptom reduction over time from Pre to Post and from Pre to FU was associated with higher levels of treatment credibility for self-referred patients.

#### 3.2.3 System related predictors

The results showed no associations between pre-treatment symptom levels and referral groups.

Primary aim: From Pre to Post and from Pre to FU, statistically significant referral-group differences in symptom change were found when accounting for both patient- and treatment-related predictors (Table 2).

The results based on z-score transformation of predictors and outcome variables showed that higher treatment credibility and being employed were the strongest predictors for the total sample.

Secondary aim: Differentiating between the referral pathways, statistically significant different magnitudes in relations were found for almost all variables (Table 3, see ∆). The differences were positive for eight relations, with higher values for the self-referred, while five differences in relations were negative. The strongest predictors of symptom reduction across the two intervals in the GP-referred group were being employed and higher levels of treatment credibility. In the self-referred group, the strongest predictors in these intervals were being a woman, lower age, higher credibility levels and lower levels of social support.

## 4 Discussion

As addressed in our primary aim, this study investigated the unique contributions of patient-, treatment-, and system-related predictors of symptom reduction in guided internet-delivered cognitive behavioral therapy (guided ICBT) for depression and anxiety disorders in a routine care clinic. Our second aim explored whether the predictive value of these factors varied depending on referral pathway by comparing patients referred by general practitioners (GP) with those who self-referred.

The overall results showed that in guided ICBT, symptom reduction over time may be associated with a complex interplay between patient-, treatment-, and system-related predictors. The predictors varied in how strongly they were associated with outcome, suggesting that treatment effectiveness depends on more than individual predictors considered in isolation. Importantly, the referral pathway itself not only predicted outcomes but also moderated the relations between the individual predictors and symptom reduction. This approach supports a more context-sensitive interpretation of treatment outcome and highlights how effects may emerge in specific subgroups when multiple predictors are considered simultaneously. Such complexity is often overlooked in studies that rely on bivariate analyses.

### 4.1 Patient-related factors

Almost all the patient-related factors showed statistically significant associations with treatment outcome, and several aligned with previous literature on predictors of symptom reduction in internet-delivered interventions. Addressing our first aim, and concerning the total sample, women showed greater reduction than men. This is consistent with prior research suggesting a small but statistically significant advantage for women in depression treatment outcomes (Andersson et al., 2019; Sextl-Plötz et al., 2024). Interestingly, women presented with higher pre-treatment symptoms than men but also decreased more and therefore reached similar post-treatment symptom level. In relation to our second aim, gender differences in symptom reduction also varied by referral pathway were self-referred women had greater improvement than men from Pre to Post and from Pre to FU. However, the gender difference was weaker among GP-referred patients at Post and reversed at FU, with men showing greater improvement than women, meaning that the gender difference was temporary.

Consistent with our primary aim, age emerged as a statistically significant predictor across the total sample when all other predictors were accounted for. This indicates that younger individuals experienced greater symptom reduction over time. This effect was also found in both referral groups separately, aligning with our secondary aim. Our results do not align with a study on self-referred patients with health anxiety, where higher age predicted treatment completion and greater symptom reduction (Österman et al., 2024). Two other studies did not find age as a significant predictor; one focused on depression (El Alaoui et al., 2016), the other on anxiety disorders (Arndt et al., 2020). All these three studies were conducted within routine care using guided ICBT. These mixed findings are consistent with conclusions from systematic reviews, highlighting age as an inconsistent and generally weak predictor (Haller et al., 2023; Sextl-Plötz et al., 2024). This variability suggests that the predictive role of age in guided ICBT within routine care may be influenced by predictors such as referral pathway and diagnostic group. It also emphasizes the need to interpret demographic predictors within their broader treatment setting.

In the total sample, education was not a predictor of symptom reduction from Pre to Post but higher education was associated with less reduction from Pre to FU. The sample specific results showed that among the GP-referred, higher education was associated with stronger symptom reduction at both assessment points, although the effect was weaker at FU. It has previously been noted that self-referral in general (Harvey-Sullivan et al., 2024), and guided internet-delivered interventions in particular regardless of referral pathway, may attract individuals with higher levels of education (Hedman et al., 2013; Hedman et al., 2014; El Alaoui et al., 2015c). This pattern of educational level is also reflected among the self-referred in our sample who generally reported higher levels of education compared to GP-referred patients. Symptom reduction among the GP-referred from Pre to Post partially contrasts with results from a machine learning-study, where greater symptom reduction was associated with not having a university degree (Hornstein et al., 2021). A similar pattern was found in a routine care study of guided ICBT, where higher education predicted lower pre-treatment symptom severity but also less change over time (Edmonds et al., 2018). While both these studies suggest that lower education may be associated with greater symptom reduction, our results indicate that higher education may facilitate both early, and long-term improvement among GP-referred patients.

Shorter duration of illness was associated with more symptom reduction from Pre to Post and Pre to FU in the total sample. When divided by referral pathway, this relation was only found in the GP-group, despite both referral groups presenting with comparable symptom severity and illness duration pre-treatment. Although exceptions may exist, duration of illness has typically not been found as a significant predictor in prior studies or reviews concerning depression and anxiety disorders (Haller et al., 2023; Sextl-Plötz et al., 2024; El Alaoui et al., 2015a, El Alaoui et al., 2016). This raises the possibility that the clinical meaning of chronicity may be context-sensitive, shaped by referral pathway.

Being employed was among the strongest predictors associated with symptom reduction from Pre to Post and from Pre to FU in the total sample. However, when exploring the second aim, this relation was only found in the GP-referred group. These findings echoes previous individual studies where being employed predicted lower post-treatment symptoms (El Alaoui et al., 2016; Hedman et al., 2012; Niles et al., 2021), whereas findings from a systematic review only partially support this (Haller et al., 2023). Notably, a significantly higher proportion of self-referred patients in our study were employed compared to the GP-referred. The difference in employment between the referral-groups may help explain why employment did not emerge as a significant predictor among the self-referred. This lack of association may potentially reflect a ceiling effect whereby limited variability in employment status reduces its explanatory power among the self-referred (Garin, 2023).

In relation to our first aim, lower pre-treatment levels of social support were associated with greater symptom reduction from Pre to Post and from Pre to FU. When exploring our second aim, the consistency of this result suggests that referral pathway did not moderate the relationship between social support and symptom reduction. Although somewhat unexpected, the finding challenges the common assumption that higher social support facilitates better outcomes. Supporting this common assumption, a large primary care study found that higher levels of support predicted greater improvement (Hallgren et al., 2017). Several factors may explain this discrepancy. Our study was conducted in specialist care and focused exclusively on guided ICBT, whereas Hallgren, Lundin (Hallgren et al., 2017) included three different interventions delivered in a primary care setting. It is also possible that patients with low social support engage more actively with the therapist-guided interventions to compensate for limited external support.

### 4.2 Treatment-related predictors

Addressing our first aim, both treatment credibility and self-efficacy predicted symptom reduction from Pre to Post, with credibility emerging as one of the strongest predictors. This supports previous findings identifying these two variables as consistent, but modest predictors of treatment outcomes (Haller et al., 2023; Kumpasoğlu et al., 2025; Pontén et al., 2024). While treatment credibility remained a consistent predictor from Pre to FU, the role of self-efficacy appeared to diminish or even reverse in this time-period, suggesting a more complex or time-limited role. This is reflected by the mixed results of self-efficacy's predictive value in internet-delivered interventions reported in a systematic review (Haller et al., 2023). When examining treatment credibility and self-efficacy by referral pathway in our second aim, both predictors demonstrated stronger associations with symptom reduction among GP-referred. In this referral-group, higher levels of both predictors were associated with greater symptom reduction at FU. In contrast, these predictors were weaker or non-significant among the self-referred. As those who self-referred reported higher pre-treatment levels of treatment credibility than the GP-referred, this may have resulted in reduced variability, potentially weakening or eliminating its association with other predictors. Another possible explanation for the referral pathway differences at FU involves treatment adherence. A systematic review of predictors of adherence in internet-delivered interventions identified higher treatment credibility as a significant factor, whereas self-efficacy was not (Beatty and Binnion, 2016). Based on this, the continued improvement among GP-referred may stem from continued engagement with the CBT-exercises after the structured intervention ended. However, as our study did not assess adherence, we are unable to directly evaluate this possibility.

### 4.3 System-related predictors

As previously reported, self-referred patients reported greater symptom reduction than GP-referred from Pre to Post and from Pre to FU (Bjarke et al., 2025). In the present study, this result remained significant even when holding constant the statistical effects of patient- and treatment-related predictors. While the GP-referred patients continued to improve after the structured intervention ended, self-referred patients reported no significant change from Post to FU. In relation to our second aim, referral pathway also moderated the associations between pre-treatment characteristics and symptom reduction, indicating that the relevance of specific predictors varied by referral-group.

The referral pathway-specific associations suggest that different mechanisms may underlie symptom reduction depending on how patients accessed care and may also reflect deeper differences in underlying patient profiles. These findings suggest that referral pathway is not merely a logistical detail but a meaningful contextual factor that may shape both who enters treatment and how they respond. In previous studies referral pathway has typically been examined as an independent predictor (Hornstein et al., 2021; White, 2009), without considering its associations with patient- or treatment-related predictors. When analyzed in isolation, system-related predictors may appear secondary. However, by analyzing system-related predictors alongside patient- and treatment-related predictors, we gain a more nuanced understanding of how context shapes treatment response. This integrated perspective suggests that system-related predictors do not simply influence access to care, it interacts with patient- and treatment-related predictors to shape the conditions under which change occurs. This integrated perspective suggests that system-related predictors are part of the therapeutic context itself, influencing both the relevance and impact of other predictors.

### 4.4 Clinical relevance

Given that guided ICBT does not yield uniform outcomes across all patients (Rozental et al., 2019; Andersson et al., 2019), our findings from routine clinical care have identified patient- and treatment-related factors that may contribute to increased symptom reduction depending on how patients accessed care. While patient-related factors like age, educational level, employment status, and social support are typically not modifiable at the point of care, treatment-related factors like self-efficacy and treatment credibility can be. These factors can be assessed and actively reinforced by referring clinicians. In this context, the clinician plays a crucial role in setting the patient up for success by fostering belief in the treatment and the patient's own capacity to engage with it. For self-referred patients, these motivational and cognitive factors may already be strong, as their active choice of treatment suggests higher pre-treatment levels. However, reinforcement may still be necessary to support long-term maintenance of therapeutic gains, particularly after the structured intervention ends. Recognizing these differences may help tailoring a more personalized treatment to optimize outcomes across referral pathways.

## 5 Limitations

A key strength of this study is its integrative approach, modeling patient-, treatment-, and system-related predictors concurrently within a relatively large routine care sample. However, there are some limitations. First, there was some attrition, particularly at the 6-month follow-up. We addressed this by EM imputation under the MAR assumption. However, this assumption cannot be empirically verified. If the missingness was systematically related to unobserved variables, this could have introduced bias into the estimates. A second limitation concerns the interpretation of duration of illness. While this variable was included, we lacked information about patients' previous treatments. Without knowing whether a longer illness duration reflects untreated symptoms, multiple failed interventions, or chronicity despite ongoing care, the clinical meaning of this variable remains ambiguous. This limits our ability to fully assess its predictive value and may have introduced unmeasured heterogeneity into the analyses. Third, all study data were collected through the same secure web platform. However, since data collection began in 2014, the platform has not kept pace with technological advancements. As a result, it does not support backend extraction of user data, which limits our ability to examine potentially valuable predictors such as digital engagement patterns and module completion speed.

## 6 Future research

The present study highlights the importance of analyzing patient-, treatment-, and system-related predictors in combination, rather than in isolation. Future research should build on this integrative approach to better understand how these domains interact to shape treatment response in guided ICBT. Rather than focusing solely on outcome differences between referral pathways, future studies should aim to identify the mechanisms through which system-level predictors interact with patient- and treatment-related predictors. This may involve exploring how patients' expectations, prior experiences with care, or contextual barriers shape their engagement and outcomes. By expanding the scope to include dynamic and contextual influences across all three predictor domains, future research can contribute to a more personalized and equitable model of internet-delivered mental health care — one that moves closer to understanding what works for whom in which context.

## 7 Conclusion

This study sought to deepen the understanding of what works for whom in which context by investigating treatment outcomes in guided ICBT for depression and anxiety disorders. It used unique and adjusted novel models that simultaneously accounted for patient-, treatment-, and system-related predictors. By analyzing these domains concurrently, the study moves beyond traditional predictor analyzes of patient characteristics and demonstrates how referral context shaped the relevance and impact of patient- and treatment-related predictors in routine care. This integrative approach offers a more context-sensitive understanding of therapeutic change and supports the development of more tailored and equitable models of internet-delivered mental health care.

## Declaration of Generative AI and AI-assisted technologies in the writing process

During the preparation of this work the main author used ChatGPT, GPT-4 in order to support grammar and readability. After using this tool, the main author reviewed and edited the content as needed and take full responsibility for the content of the publication.

## Role of funding sources

This study was part of the Center for research-based innovation on Mobile Mental Health (ForHelse), which is funded by the 10.13039/501100005416Norwegian Research Council (NFR: 309264).

## Declaration of competing interest

The authors declare that they have no known competing financial interests or personal relationships that could have appeared to influence the work reported in this paper.

## Figures and Tables

**Table:** 

**Statement**	**1**	**2**	**3**	**4**
I do not feel strongly involved in anyone else's life	☐	☐	☐	☐
I do not know anyone where I live who would spend several hours helping me with an important task	☐	☐	☐	☐
I do not know anyone in my neighborhood who could cook or shop for me if I were ill	☐	☐	☐	☐
I feel emotionally close to others	☐	☐	☐	☐
If I needed a friend to help with renovations, I have someone where I live who would help me	☐	☐	☐	☐
I do not know many people who genuinely care about me	☐	☐	☐	☐

**Table 1:** Percentage changes in observed diagnosis-specific symptom scores from Pre to Post and from Pre to FU for the total sample and for both referral pathways in depression, panic disorder, and social anxiety disorder.

		Total		GP		Self		Difference
		%		%		%		p
Depression: MADRS	Post	31.7		14.7		50.4		.001
	FU	43.0		30.1		55.3		.020
Panic disorder: BSQ	Post	30.7		28.1		35.6		.055
	FU	39.5		35.0		47.3		.017
Social anxiety disorder: SPS	Post	31.8		28.2		40.4		.151
	FU	42.6		37.7		49.7		.141

Pre: pre-treatment, Post: post-treatment, FU: 6-month follow-up

MADRS-S: Montgomery Åsberg Depression Rating Scale, Self-rating version, BSQ: Body Sensation Questionnaire, SPS: Social Phobia Scale.

Self: self-referred, GP: referred by General Practitioner

## Data Availability

This section collects any data citations, data availability statements, or supplementary materials included in this article.

## Appendix 1 Social Support – Questionnaire

1. How many people are so close to you that you can count on them if you have personal problems?
  ☐ 0 ☐ 1–2 ☐ 3–5 ☐ 6 or more

2. The statements below describe people in different ways. For each item, choose the number that best matches your assessment of yourself:
  1 = completely true for me 2 = partly true for me 3 = partly not true for me 4 = not at all true for me
